# Structural characterization and *in vitro* myocardial injury protection potential of one acidic polysaccharide from blueberry (*Vaccinium spp*.) pomace

**DOI:** 10.3389/fnut.2026.1920321

**Published:** 2026-09-22

**Authors:** Beibei Zhang, Qiwen Zhang, Ning Huang, Mengmeng Jia, Han Xing, Yongjie Yang, Dinglei Xue, Jing Yang, Zifei Qin

**Affiliations:** 1 Department of Pharmacy, Henan Province Engineering Research Center of Application & Translation of Precision Clinical Pharmacy, The First Affiliated Hospital of Zhengzhou University, Zhengzhou, China; 2 Jiangsu Sanshu Biotechnology Co., Ltd., Nantong, China

**Keywords:** acidic pectin polysaccharide, blueberry pomace, myocardial injury protection potential, PI3K/AKT/mTOR and MAPK pathways, structural characterization

## Abstract

**Introduction:**

Blueberry pomace is the by–product of blueberry (*Vaccinium spp*.) fruits. This study aimed to prepare blueberry pomace polysaccharide (BBPP) and explore its myocardial injury protection potential.

**Methods:**

An acidic polysaccharide (BBPP80–1a) was isolated and purified from active BBPP80-1 fraction. Its structure was characterized based on some approaches. Its myocardial injury protection potential was performed in hypoxia/reoxygenation (H/R)-induced H9c2 cells injury model.

**Results:**

The molecular weight of BBPP80–1a was 164.91 kDa. The ratio of galacturonic acid (GalA) was 50.92% in methylation results. Further, nuclear magnetic resonance data suggested repetitive → 4)-α-D-Gal*p*A-(1 → and → 4)-α-D-Gal*p*A-6-OMe-(1 → residues occupied the homogalacturonan region of backbone. It had a highly porous, reticular, and heterogeneous microstructures. Moreover, BBPP80–1a can obviously protect H9c2 cells from H/R injury *via* phosphoinositide 3-kinase / protein kinase B / mammalian target of rapamycin (PI3K/AKT/mTOR) and *c*-Jun *N*-terminal kinase / mitogen-activated protein kinase (JNK/MAPK) pathways.

**Discussion:**

BBPP80–1a is a pectic polysaccharide. It exhibited the potential on cardioprotective effect through the activation of PI3K/AKT/mTOR survival pathway and the regulation of JNK/MAPK pathways.

## Graphical Abstract

**Figure:**
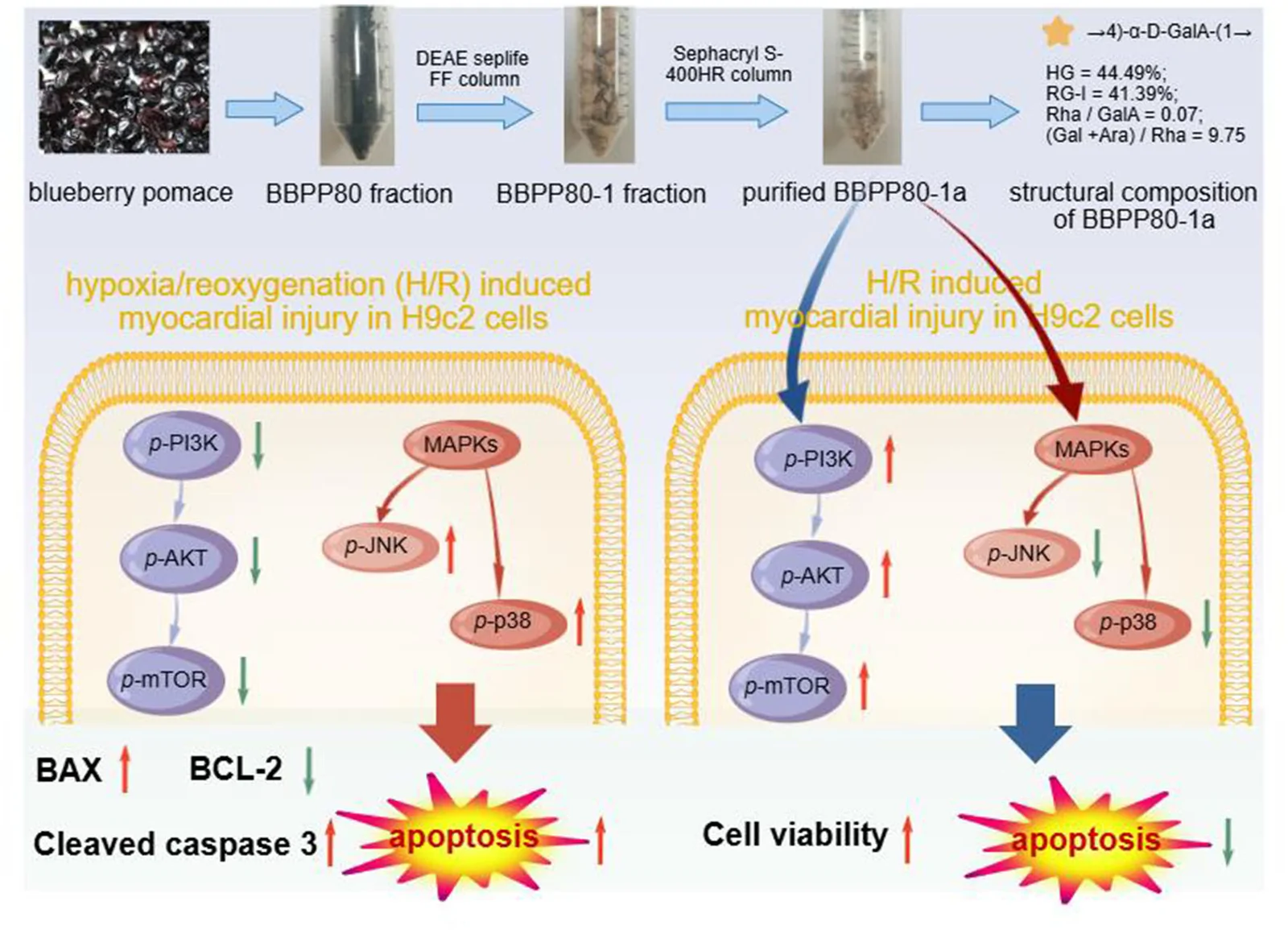

## Highlights

1. Polysaccharide preparation is an attempt to utilization of blueberry pomace.
2. BBPP80–1a is a pectic polysaccharide from BBPP80–1 active fraction.
3. BBPP80–1a polysaccharide can improve H/R-induced cell apoptosis.
4. BBPP80–1a exerted cardioprotective effects through PI3K/AKT/mTOR and JNK/MAPK pathways.

## Introduction

Cardiovascular diseases have become a leading cause of mortality worldwide (1, 2). Myocardial apoptosis induced by ischemia/reperfusion (I/R) injury is one of the most important pathogeneses of myocardial infarction and heart failure (3, 4). Although reperfusion therapy is the standard treatment method for restoring myocardial blood flow, it inevitably triggers oxidative stress and inflammation, ultimately leading to cardiomyocyte death and poor prognosis (5, 6). Therefore, it is necessary to identify the effective therapeutic strategies that can significantly alleviate I/R injury and inhibit cardiomyocyte apoptosis. Recently, clinical trials and pharmacological experiments have shown that some traditional Chinese medicine prescriptions (TCMP) have definite therapeutic effects on anti-myocardial ischemia, improving myocardial no reflow and reperfusion injury (7–11). It is of great significance for developing novel natural cardiovascular protective agents. Polysaccharides are the most abundant component of many plants, herbal medicines and foods (12, 13). They have got considerable attention in drug discovery and functional food development due to their low toxicity, high biocompatibility, and multi-target regulatory properties (14, 15). Notably, some polysaccharides possess cardioprotective effects through improving PI3K/AKT and MAPK pathways, highlighting their potential as promising candidates for treating myocardial I/R injury (3, 16, 17).

Blueberries (*Vaccinium spp*.) are widely recognized for their cardiovascular benefits due to the rich contents of bioactive compounds. For example, a 6-month, double-blind, randomized controlled trial indicated that the sustained improvements in vascular function, lipid status, and underlying NO bioactivity of patients with metabolic syndrome following 1 cup blueberries per day (18). Polysaccharides are one class of the most important active components of blueberries. Previous study showed that BLP-2 and BLP3 polysaccharides from blueberry leaf exhibited high bile acid binding activity and α-amylase inhibitory activity, indicating their potential for lowering blood lipids and blood glucose levels (19). BFP3 from blueberry fruit exhibited good lipid-lowering activity with binding rates of 79.95% and 78.50% for sodium glycocholate and sodium taurocholate, respectively (20). During consumption or processing of blueberry, the pomace which accounts for approximately 25–30% of the fruit were discarded as a low value by–product (16). This brings huge resource waste and environmental pollution (16). Many reports had illustrated that polysaccharides from other plant or fruit by-products (red grape pomace, *Polygonati rhizoma* pomace, date pomace, etc.) demonstrated significant lipid-lowering, anti-inflammatory, and cardioprotective effects (21). Blueberry pomace is not only rich in dietary fiber and phenolic compounds, but contains abundant active polysaccharides, representing a renewable biological resource with great development potential (16). In our previous study, the refined crude polysaccharide from blueberry pomace can significantly reduce the body weight, serum lipid level and aortic plaque formation of atherosclerosis mouse model (16). Further, one purified neutral polysaccharide (BBPP80–2a) and an acidic polysaccharide (BBPP80–2b) both can protect H9c2 cardiomyocytes from hypoxia/reoxygenation (H/R)–induced apoptosis by improving PI3K/AKT/mTOR and JNK/ERK/MAPK pathways (16). These findings preliminarily revealed the myocardial injury protection potential of polysaccharides derived from blueberry pomace. The structures and underlying mechanism of bioactive polysaccharides in blueberry pomace are worth further exploration.

Accumulating evidence indicates that plant polysaccharides can inhibit the apoptosis by activating PI3K/AKT/mTOR pathway (17, 22, 23). Meanwhile, JNK/MAPK pathway also participates in oxidative stress and cellular injury responses, with aberrant MAPK pathway activation promoting apoptosis (24–26). In previous study, the structure–activity relationship of purified blueberry pomace polysaccharides toward cardioprotection remained to be further elucidated (16). The diversity in structures may be the important factor in determining the biological activities of polysaccharides (27, 28). For example, molecular weight, monosaccharide composition, glycosidic linkage type, and advanced conformation all can influence their interaction with biological targets (29, 30). Pectin polysaccharides are also important components of blueberry pomace (31). The characteristic units including galacturonic acid and the rhamnogalacturonan–I (RG–I) domain in pectin polysaccharides are often considered to be corresponding to immune regulation and antioxidant activity (32, 33). Except BBPP80-2 fraction, BBPP80-1 fraction also occupied most of blueberry pomace crude polysaccharides (16). Therefore, it is necessary to prepare more purified polysaccharides from blueberry for deeper exploration of structure-activity relationships and developing them into novel functional agents.

In the present study, we hypothesized that BBPP80–1 fraction and its-rich purified polysaccharide can reduce apoptosis and exert cardioprotective activity through PI3K/AKT and MAPK pathways. Therefore, the analytical flowchart for structural characterization and *in vitro* myocardial injury protection potential of one acidic polysaccharide was shown in Figure 1. First, the protective activity of BBPP80–1 fraction was conducted in H/R-induced H9c2 myocardial cell injury model. In addition, a novel acidic pectic polysaccharide (BBPP80–1a) was purified from active BBPP80–1 fraction. Its structure was elucidated based on molecular weight determination, monosaccharide composition analysis, methylation and gas chromatography–mass spectrometry (GC–MS) analysis, nuclear magnetic resonance (NMR) data, and scanning electron microscopy (SEM) analysis. Moreover, the underlying protective mechanism involving in cell viability, apoptosis rate, and protein expression of key targets in BCL-2/BAX, PI3K/AKT/mTOR and JNK/MAPK pathways in H/R-induced H9c2 cell injury model were systematically explored. This study would benefit the known about the function of polysaccharides in blueberry pomace, and providing a new pathway for the high-value utilization of blueberry pomace.

**Figure 1:**
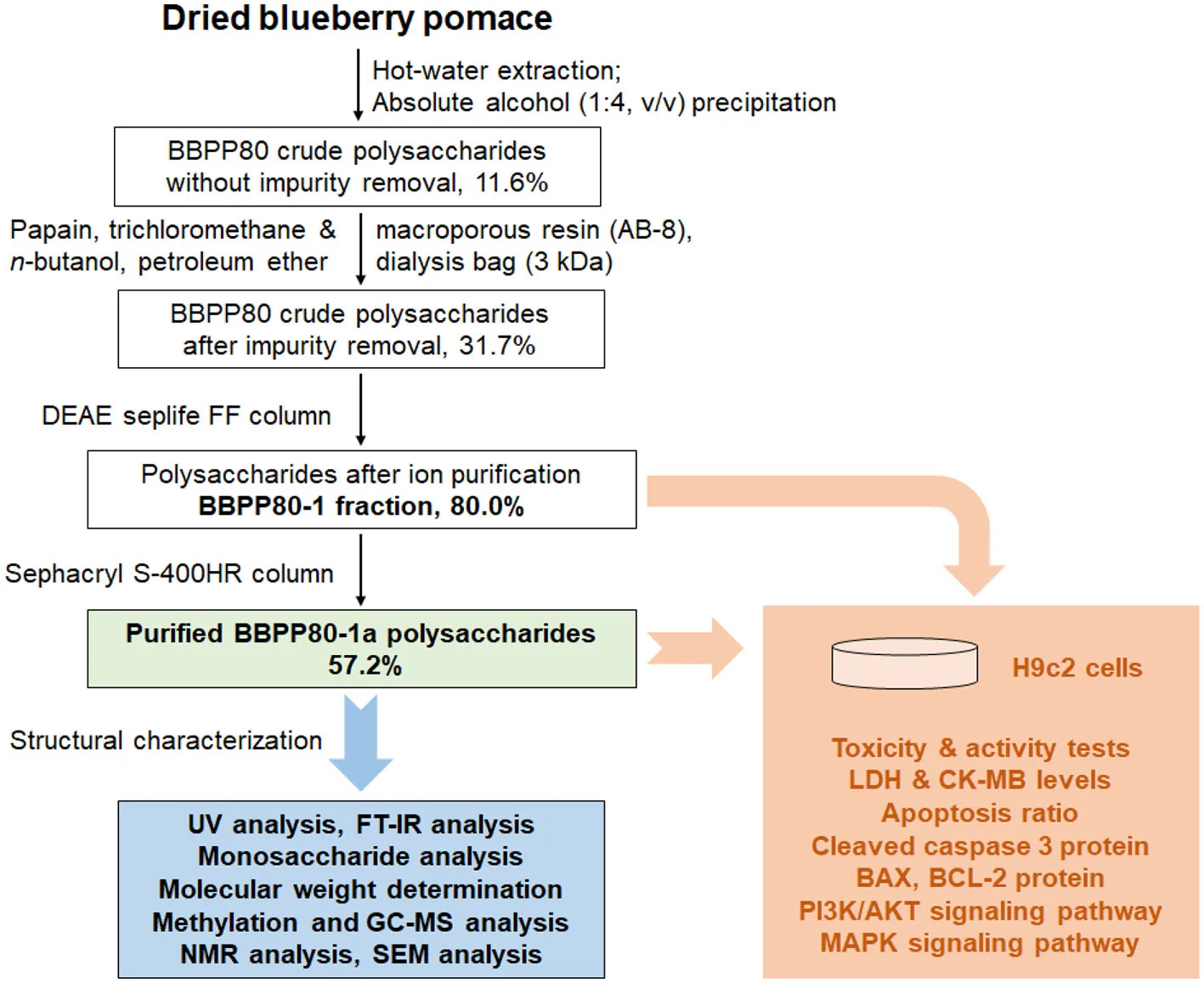
The flowchart for structural characterization and *in vitro* myocardial injury protection potential of one blueberry pomace acidic polysaccharide.

## Materials and method

### Materials and reagents

Blueberry samples were sourced from Baishan city of Jilin province in China. H9c2 cells were obtained from Shanghai Guandao Bioengineering Co., Ltd (Shanghai, China). Each monosaccharide standard was were acquired from Sigma-Aldrich (St. Louis, Missouri, USA). LY294002 (#LY294002), SP600125 (#SP600125) and SB203580 (#SB203580) were all purchased from Selleck Chemicals LLC (Houston, TX, USA). Creatine kinase-MB isoenzyme (CK-MB) and lactate dehydrogenase (LDH) kits were purchased from Zhengzhou Chuangsheng Bioengineering Co., Ltd (Zhengzhou, China). The cleaved caspase-3 antibody (ab32042) was sourced from ABCAM plc (Cambridge, Massachusetts, USA). Antibodies for BAX (#2774), BCL-2 (#3498), AKT (#9272S), *p*-AKT (#9271S), mTOR (#2972S), and *p*-mTOR (#5536) were all obtained from Cell Signaling Technology (Boston, USA). Antibodies for PI3K (#AF6242), and *p*-PI3K (#AF3242) were purchased from Affinity Biosciences plc (Melbourne, Australia). The MAPK (#AM076) antibody was procured from Beyotime Biotechnology Co., Ltd. (Shanghai, China). The *p*-MAPK (#BN41104R) antibody was sourced from Beijing Biorigin Biotechnology Co., Ltd. (Beijing, China).

### Extraction, isolation and purification of BBPP80–1a polysaccharide

First, the blueberry pomace was dried and crushed to powder, and filtered with a 60-mesh sieve. The samples were soaked with absolute ethanol solution overnight for degreasing and decolorization, respectively. After centrifugation at 6,000 rpm for 10 min, the precipitate was collected and further extracted by hot-water (w/v, 1:10) for 4 h at 60°C twice. The combined extract solutions were concentrated to a certain volume under vacuum evaporate conditions. Absolute ethanol (v/v, 1:4) was added overnight at 4°C for alcohol precipitation. After centrifugation at 8,000 rpm for 10 min, the precipitated solid was collected and it was blueberry pomace crude polysaccharides (BBPP80).

The BBPP80 crude polysaccharides were fully re-dissolved in water, and papain was added to perform enzymatic hydrolysis overnight. About 1/4 volume of chloroform and *n*-butanol was added to the aqueous phase. The mixture was mixed thoroughly and the upper aqueous phase was collected. Petroleum ether was further added to the liquid and mixed thoroughly. The lower aqueous phase was collected. Macroporous resin AB-8 was added to the aqueous phase, mixed thoroughly, and placed overnight. The liquid was collected and dialyzed in a 3 kDa dialysis bag for 48 h to remove small molecular components. The solution was freeze dried to obtain the refined BBPP80 crude polysaccharides.

Refined BBPP80 samples were separated on a diethylaminoethyl (DEAE) cellulose column (Φ26 mm × 400 mm) with NaCl solutions of 0, 0.1, 0.2, and 0.3 mol/L as the eluent at a flow rate of 4 mL/min. Each fraction was collected and assayed for the carbohydrate content by Phenol–sulfuric acid method at 490 nm. The main polysaccharide fractions were collected and concentrated. The residue was dissolved with distilled water and dialyzed (3 kDa) for desalination for 48 h. Different polysaccharide-enriched eluent was collected and concentrated to obtain BBPP80–1 fraction. Further, BBPP80–1 sample was loaded and separated on a Sephacryl S-400 column (Φ26 mm × 1,000 mm), eluted with distilled water at a flow rate of 1.0 mL/min, and monitored by the Phenol -sulfuric acid method. Selected eluents were collected and concentrated to prepare purified BBPP80–1a polysaccharide.

### Structural characterization of BBPP80–1a polysaccharide

#### UV–Vis and FT-IR analysis

BBPP80–1a sample was dissolved in water solution at a concentration of 5 mg/mL. The UV–vis spectrum was performed by a Thermo Fisher Scientific Multiskan GO microplate reader at 200–800 nm. Pure water under the same processing conditions was used as blank control.

In addition, BBPP80–1a sample (3 mg) was mixed with KBr (200 mg), and the mixture was pressed into a 1 mm pellet. The pellet was tested using the Thermo Fisher Scientific Nicolet iZ-10 spectrometer for FT-IR measurement. The data was recorded in the range of 4,000 to 400 cm^−1^ with a resolution of 4 cm^−1^.

#### Molecular weight determination

The homogeneity and molecular weight of various fractions were measured using size exclusion chromatography multi-angle laser light scattering refractive index (SEC-MALLS-RI). A DAWN HELEOS II laser photometer (Wyatt Technology, USA) was used to measure the parameters including the weight-average molecular weight (*M*_w_), number-average molecular weight (*M*_n_), polydispersity index (*M*_w_/*M*_n_), and so on.

BBPP80–1a samples were dissolved in 0.1 M NaNO_3_ solution containing 0.02% NaN_3_ (w/w) at a final concentration of 1 mg/mL. Before use, the solutions were both filtrated with 0.45 μm filter membrane. Two series-connected Shodex Ohpak SB-805 HQ (300 × 8 mm) and Ohpak SB-803 HQ (300 × 8 mm) columns were applied to separate BBPP80–1a samples at 45 C with the flow of 0.6 mL/min. The mobile phase A was 0.1 M NaNO_3_ solution containing 0.02% NaN_3_ (w/w) with equal gradient elution for 75 min. The injection volume was 100 μL.

Further, BBPP80–1a samples were dissolved in DMSO solution containing 0.5% lithium bromide (w/w) at a final concentration of 1 mg/mL. Before use, the solutions were both filtrated with 0.45 μm filter membrane. Three series-connected Ohpak SB-805 HQ (300 × 8 mm), Ohpak SB-804 HQ (300 × 8 mm), and Ohpak SB-803 HQ (300 × 8 mm) columns were used to separate BBPP80–1a samples at 60 C with the flow of 0.3 mL/min. The mobile phase B was DMSO solution containing 0.5% lithium bromide (w/w) with equal gradient elution for 120 min. The injection volume was also 100 μL, and three replicate measurements were performed.

The sample concentration and *d*_n_/*d*_c_ values were monitored on differential refractive index detector (Optilab T-rEX, Wyatt Technology, USA). The *d*_n_/*d*_c_ values of the samples for 0.1 M NaNO_3_ containing 0.02% NaN_3_ and DMSO solutions were 0.141 and 0.07 mL/g, respectively. ASTRA 6.1 software was employed to process the data.

#### Monosaccharide composition analysis

BBPP80–1a samples were treated with 1 mL of 2 M TFA solution at 121 C for 2 h. The samples were dried and washed with absolute methanol three times. The residue was dissolved in sterile water, filtered through 0.22 μm membrane, and analyzed on a Dionex CarboPac PA-20 anion-exchange column (150 × 3.0 mm, 10 μm) at 30 C with a flow rate of 0.5 mL/min by high-performance anion-exchange chromatography (HPAEC) (Dionex ICS 5000+ system, Thermo Fisher Scientific, USA) with a pulsed amperometric detector. The mobile phases consisted of H_2_O (A), 0.1 M NaOH solution (B), and 0.1 M NaOH solution containing 0.2 M NaAc (C). The elution gradient was as follows: at 0–min, A/B/C (95:5:0, v/v/v); at 26–min, A/B/C (85:5:10, v/v/v); at 42–min, A/B/C (85:5:10, v/v/v); at 42.1–min, A/B/C (60:0:40, v/v/v); at 52–min, A/B/C (60:40:0, v/v/v); at 52.1–min, A/B/C (95:5:0, v/v/v); at 60–min, A/B/C (95:5:0, v/v/v). The injection volume was 5 μL, and three replicate measurements were performed. Chromeleon 7.2 Chromatography Data System (Thermo Scientific) was used to process the acquired data.

#### Methylation and GC-MS analysis

The reduction of uronic acids of BBPP80–1a was performed by using the method of methylation. BBPP80–1a samples (5 mg) were dissolved in water, and reacted with 1 mL of 100 mg/mL of (1-cyclohexyl-3-(2-morpholinoethyl)-carbodiimide metho-*p*-toluenesulfonate) solution for 2 h. The solution was added with 1 mL of 2 M of imidazole solution. The mixture was divided into two equal parts, and treated with 1 mL of NaBH4 and NaBD4 solution (both 30 mg/mL) for 3 h, respectively. The reaction was terminated by 100 μL of glacial acetic acid. After 48 h of dialysis, the solution was freeze dried. The dried sample was dissolved in 500 μL of DMSO, and 1 mg of NaOH was added for incubation for 30 min. Further, 50 μL of CH_3_I solution was added to react for 1 h. And 1 mL of water and 2 mL of dichloromethane was supplemented into the mixture. The mixture was subjected to vortex mixing and then centrifuged. The aqueous phase was discarded. The washing process was repeated with water three times. The lower dichloromethane phase was collected and dried with nitrogen gas. In addition, 100 μL of 2 M TFA solution was used to hydrolyze the methylated derivatives at 121°C for 1.5 h. The solution was steam dried at 30°C. About 50 μL of 2 M ammonia solution and 50 μL of 2 M NaBD4 solution were added to the mixture for reaction at room temperature for 2.5 h. Further 20 μL of acetic acid was supplemented to terminate the reaction. The solution was dried with nitrogen, and washed twice with 250 μL of methanol. Acetic anhydride solutions (250 μL) were added for reaction at 100°C for 2.5 h. 1 ml of water was added and placed for 10 min. 500 μL of dichloromethane was added. The mixture was vortexed, centrifuged, and the aqueous phase was discarded. The solution was washed with water three times. Finally, the lower dichloromethane phase was taken for GC-MS analysis.

The samples were separated on an Agilent TG-200 chromatographic column (30 m × 0.25 mm × 0.25 μm, SGE, Australia) by Agilent 6890A-5977B system. The carrier gas was high-purity helium with a split ratio of 10:1. The injection volume was 1 μL, and three replicate measurements were performed. The flow rate was 1.5 mL/min. The gradient for column temperature was as follows: at 0–2 min, 150°C; then increased to 210°C at a rate of 2°C/min for 3 min; increased to 240°C by 2°C/min for 5 min. An electron bombardment ion source (EI) was used in this study. The full scan ranges (*m/z*) were set from 30 to 600 Da. The ion source temperature was 200°C, the MS quadrupole temperature was 110°C, the ionization energy was 50 eV, the transmission line temperature was 210°C. The data was processed under a MassHunter workstation.

#### NMR analysis

BBPP80–1a polysaccharide was dissolved in 0.5 mL of D_2_O to a final concentration of 40 mg/mL. The samples were measured at 25 °C by a Bruker AVANCE NEO 600 MHz spectrometer (Bruker, Rheinstetten, Germany). The pulse program for ^1^H and ^13^C were zg30 and zgpg30, respectively. The numbers of scan for ^1^H, ^13^C, ^1^H-^1^H COZY, NOESY, HMBC, and HSQC spectra were 128, 5,000, 8, 24, 52, and 18, respectively. The spectral width for ^1^H and ^13^C were 7.16, and 12.42, respectively. The relaxation delay for ^1^H, ^13^C, ^1^H-^1^H COZY, NOESY, HMBC, and HSQC spectra were 1.0, 2.0, 2.0, 2.0, 1.5, and 1.5, respectively. The ^1^H and ^13^C spectra as well as ^1^H-^1^H COZY, NOESY, HMBC, and HSQC spectra were all collected.

#### SEM analysis

BBPP80–1a sample was fixed onto a sample stub and gold-coated using a sputter coater. Its surface morphology was observed by a scanning electron microscope (SEM) (Zeiss Merlin Compact SEM, Germany). The magnifications were set at 100 × and 10,000 × under high vacuum conditions at 1.0 kV. Zeiss ZEN Connect software was used to analyzed the information of BBPP80–1a.

### Myocardial injury protection potential of BBPP80–1 and BBPP80–1a

#### Cell culture

The assays were similar with previous studies (3, 16). In brief, H9c2 cells damage model induced by hypoxia/reoxygenation (H/R) were developed in an ESCO tri-gas incubator. H9c2 cells were cultured in DMEM medium containing 10% fetal bovine serum and 0.1% antibiotic mixture. The medium was replaced and cultured with different concentrations of BBPP80–1 (or BBPP80–1a) and different inhibitors (LY294002, 10 μM; SP600125, 10 μM; or SB203580, 5 μM) solutions for 3 h. These cells were transferred to a hypoxia chamber with a gas composition of 95% N_2_ and 5% CO_2_, and cultured for 12 h. Then, these cells were returned to a standard incubator and cultured for 4 h. The absorbance of each well at 490 nm were measured to assess the cell viability.

#### Biomarkers levels

After the cells in different groups above were collected, the supernatant (100 μL) was collected to measure the levels of CK-MB and LDH at 450 and 340 nm using a COBAS INTEGRA 400 plus fully automated biochemical analyzer (Roche, Switzerland).

#### Quantification of cellular apoptosis

The H9c2 cells in different groups above were transferred into EP tubes and washed by PBS solution twice. Roche Annexin V-FLUOS kit was used to quantify the proportion of apoptosis. First, the apoptotic cells were labeled after the cells were incubated with Annexin V and propidium iodide (PI) in the dark for 15 min. A Beckman Coulter CytoFLEX flow cytometer was used to measure the proportion of apoptotic cells to determine the protective effects of BBPP80–1 fraction and BBPP80–1a polysaccharide.

#### Western blotting analysis

Bicinchoninic acid assay was used to determine the protein concentration in cell lysates. The denatured protein in different groups was loaded onto a 12% SDS-PAGE gel for electrophoretic separation. The proteins were successfully transferred to a 0.22 μM PVDF membrane. Then, the membrane was blocked with PBS solution containing 5% BSA and 0.1% Tween 20 for 1.5 h. After blocking, the membrane was co-incubated with primary antibodies (including cleaved caspase-3, BAX, BCL-2, PI3K, *p*-PI3K, AKT, *p*-AKT, mTOR, *p*-mTOR, p38-MAPK, *p*-p38-MAPK, and GAPDH) overnight at 4°C. Finally, corresponding secondary antibodies were used to co-incubate the membrane for 2 h. Visualized images and quantitative analysis were performed by Image Lab software (Bio-Rad, Shanghai, China) and ImageJ software, respectively.

#### Statistical analysis

Statistical analysis was performed using One-way ANOVA test by GraphPad Prism V5 software. *p* < 0.05 was considered to indicate statistical significance.

## Results

### Preparation of purified BBPP80–1a

The analytical process of this study was displayed in Figure 1. First, the crude polysaccharides (BBPP80 fraction) were obtained from dried blueberry pomace with yield of 11.6%. After impurities removal and dialyzed with dialysis bag of 3.0 kDa, refined BBPP80 crude polysaccharides were prepared with yield of 31.7%, and purity of 65.7%.

In addition, refined BBPP80 fraction was loaded and separated on a DEAE seplife FF column to purify the crude polysaccharides. BBPP80–1 fraction (Figure 2A) was collected with yield of 80.0%, and purity of 70.8%. Further, a sephacryl S-400HR column was used to purify BBPP80–1 fraction, and BBPP80–1a (Figure 2B) was obtained with yield of 57.2%, and purity of 91.7%.

**Figure 2:**
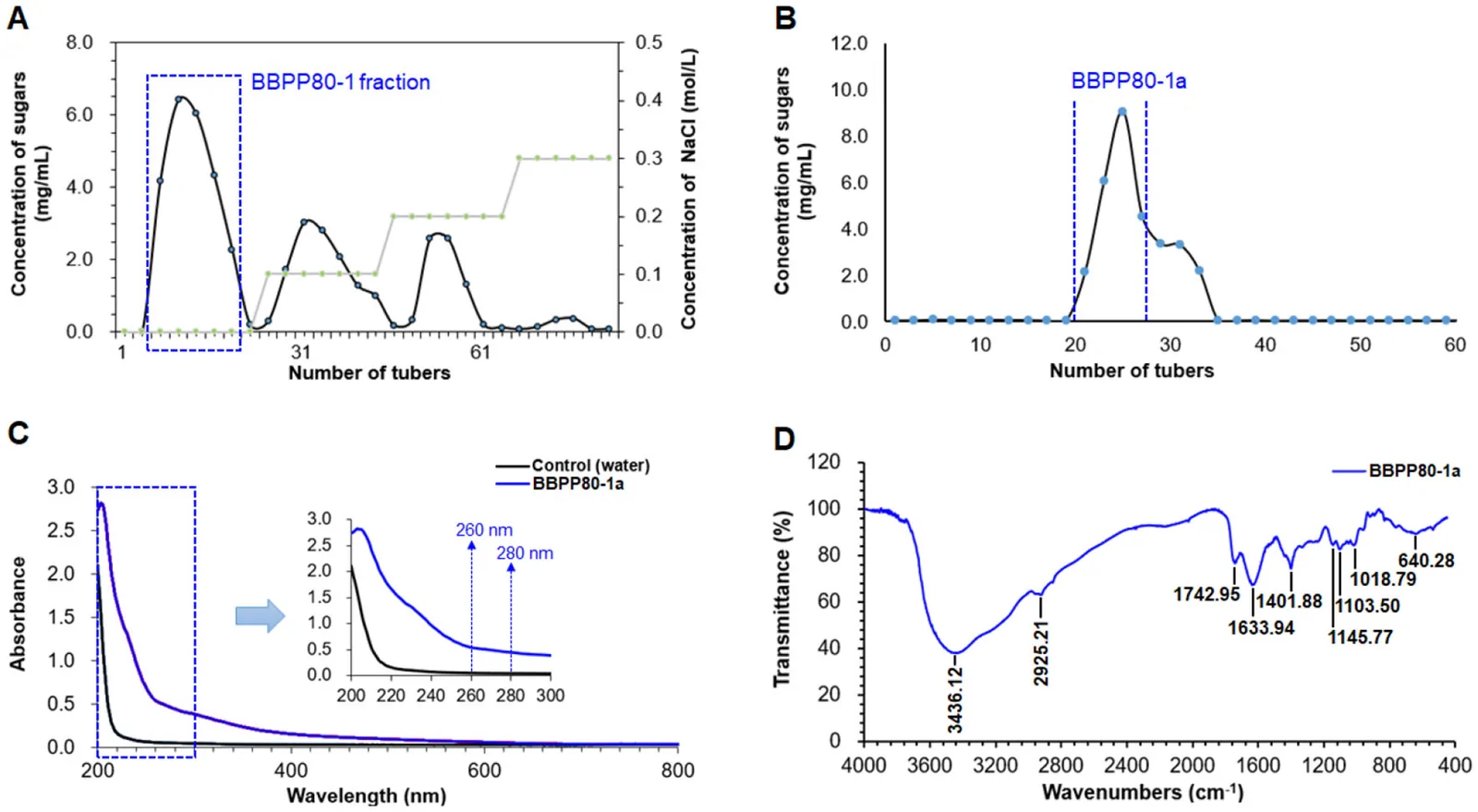
The isolation, purification, and preliminary structural characterization of BBPP80-1a polysaccharide. **(A)** Elution profile of BBPP80-1 fractions on a DEAE cellulose column; **(B)** elution profile of BBPP80–1a on a Sephacryl S-400HR column; **(C)** UV–vis spectrum of BBPP80–1a polysaccharide; **(D)** FT-IR spectrum of purified BBPP80–1a.

### Structural characterization of BBPP80–1a

The UV–vis spectrum of BBPP80–1a showed no obvious absorption peaks at 260, 280, and 620 nm, suggesting low or undetectable levels of nucleic acids, proteins, and pigments (Figure 2C). The band at 1,633.94 cm^−1^ (Figure 2D) corresponded to the asymmetric stretching of carboxyl groups (-COOH or -COO^−^), while the band at 1,401.88 cm^−1^ (Figure 2D) was considered as the symmetric stretching vibration of carboxyl groups (-COOH or -COO^−^) (34). Its *M*_w_ and *M*_n_ distributions were 164.91 and 78.43 kDa, respectively (Supplementary Figure S1; Supplementary Table S1).

The ion chromatogram (Supplementary Figure S2) and quantitative results (Supplementary Table S2) suggested that galacturonic acid and galactose were the most abundant monosaccharides which accounted for 48.01% and 22.29%, respectively. The GC–MS chromatogram (Figure 3) and mass spectrum (Supplementary Figure S3) indicated that the → 4)-Gal*p*A-(1 → unit was the most abundant sugar residue with molar ratio of 43.62%, while → 4)-Gal*p*-(1 → accounted for about 17.06% (Table 1).

**Figure 3:**
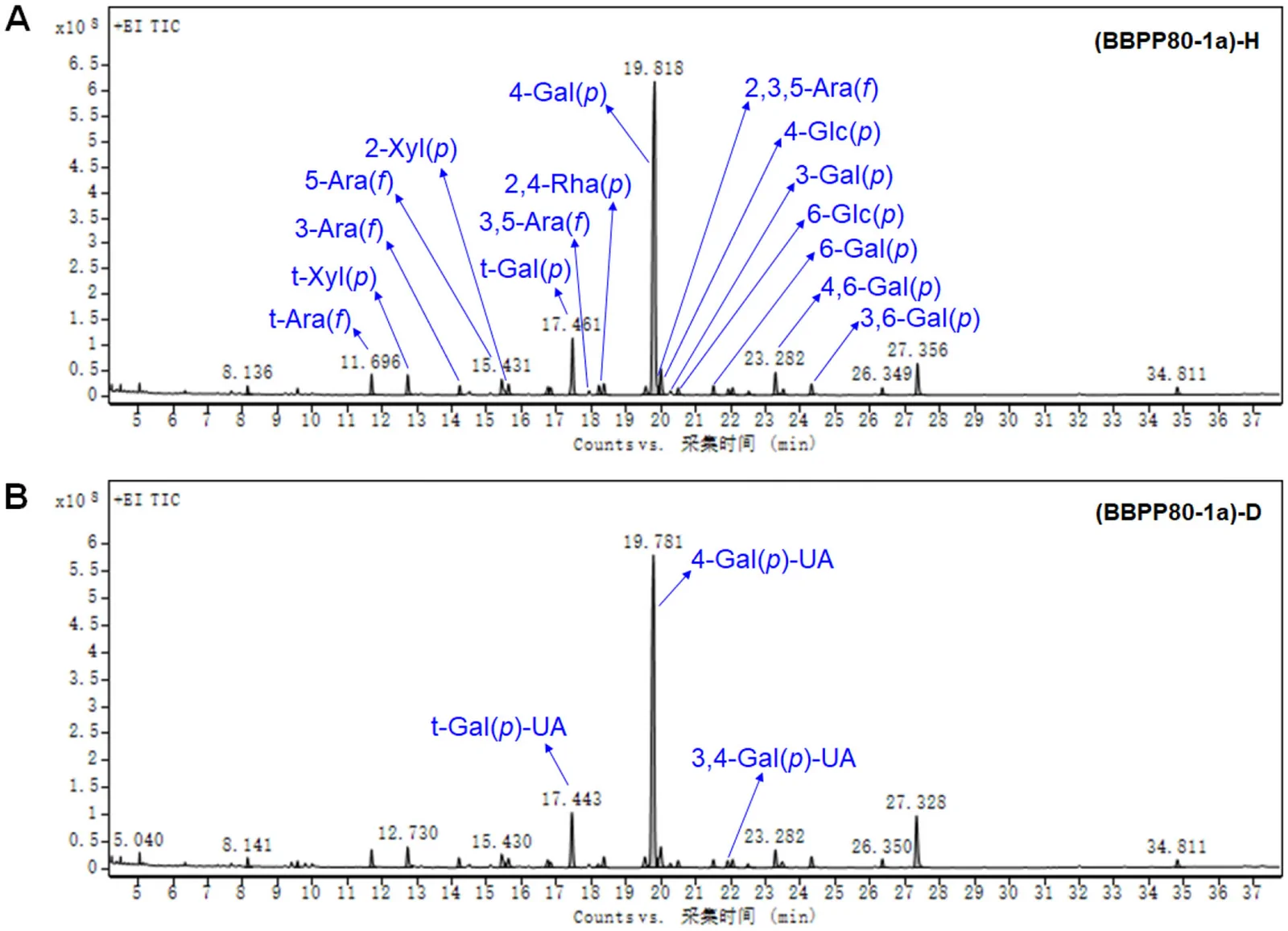
The methylation analysis of BBPP80–1a polysaccharide. **(A)** GC–MS analysis of monodeuterated BBPP80–1a sample, referring to carboxyl groups pre-reduced with sodium borohydride (NaBH4) and secondary reduced with sodium borodeuteride (NaBD4) after methylation; **(B)** the total ion chromatogram of double deuterated BBPP80–1a sample, refers to carboxyl groups reduced with NaBD4 before and after methylation.

**Table 1:** The methylation analysis results of BBPP80–1a polysaccharide by GC–MS.

No.	RT (min)	Linkage pattern	Methylated sugar	MW (Da)	Ratio (%)	Mass fragments (*m/z*)
1	11.70	Ara*f*-(1 →	1,4-di-O-acetyl-2,3,5-tri-O-methyl arabinitol	279	2.83	71,87,102,118,129,145,161
2	12.73	Xyl*p*-(1 →	1,5-di-O-acetyl-2,3,4-tri-O-methyl xylitol	279	3.77	88,101,102,118,119,161,162
3	14.51	→ 3)-Ara*f*-(1 →	1,3,4-tri-O-acetyl-2,5-di-O-methyl arabinitol	307	1.04	87,99,113,118,129,201,233
4	15.43	→ 5)-Ara*f*-(1 →	1,4,5-tri-O-acetyl-2,3-di-O-methyl arabinitol	307	2.84	87,102,118,129,162,189
5	15.62	→ 2)-Xyl*p*-(1 →	1,2,5-tri-O-acetyl-3,4-di-O-methyl xylitol	307	1.73	88,101,117,129,130,161,190
6	17.44	Gal*p*A-(1 →	1,5-di-O-acetyl-2,3,4,6-tetra-O-methyl galactitol	325	5.37	73,89,102,118,147,162,163,207
7	17.46	Gal*p*-(1 →	1,5-di-O-acetyl-2,3,4,6-tetra-O-methyl galactitol	323	3.71	87,102,118,129,145,161,162,205
8	17.94	→ 3,5)-Ara*f*-(1 →	1,3,4,5-tetra-O-acetyl-2-O-methyl arabinitol	335	1.16	85,99,118,127,159,201,261
9	18.36	→ 2,4)-Rha*p*-(1 →	1,2,4,5-tetra-O-acetyl-6-deoxy-3-O-methyl rhamnitol	349	1.68	88,101,117,130,143,190,203
10	19.78	→ 4)-Gal*p*A-(1 →	1,4,5-tri-O-acetyl-2,3,6-tri-O-methyl galactitol	353	43.62	87,99,102,115,118,131,162,175,235
11	19.82	→ 4)-Gal*p*-(1 →	1,4,5-tri-O-acetyl-2,3,6-tri-O-methyl galactitol	351	17.06	87,102,113,118,129,162,233
12	19.92	→ 2,3,5)-Ara*f*-(1 →	1,2,3,4,5-penta-O-acetyl arabinitol	363	1.19	85,86,103,115,116,128,145,146, 159,188,201,218,290
13	19.99	→ 4)-Glc*p*-(1 →	1,4,5-tri-O-acetyl-2,3,6-tri-O-methyl glucitol	351	3.17	87,102,113,118,129,162,233
14	20.27	→ 3)-Gal*p*-(1 →	1,3,5-tri-O-acetyl-2,4,6-tri-O-methyl galactitol	351	0.81	87,101,118,129,161,202,234
15	20.49	→ 6)-Glc*p*-(1 →	1,5,6-tri-O-acetyl-2,3,4-tri-O-methyl glucitol	351	1.20	87,99,102,118,129,162,189,233
16	21.50	→ 6)-Gal*p*-(1 →	1,5,6-tri-O-acetyl-2,3,4-tri-O-methyl galactitol	351	1.57	87,99,102,118,129,162,189,233
17	21.91	→ 3,4)-Gal*p*A-(1 →	1,3,4,5-tetra-O-acetyl-2,6-di-O-methyl galactitol	381	1.19	87,118,131,143,185,205,307
18	23.28	→ 4,6)-Glc*p*-(1 →	1,4,5,6-tetra-O-acetyl-2,3-di-O-methyl glucitol	379	4.33	85,102,118,127,159,162,201,261
19	24.32	→ 3,6)-Gal*p*-(1 →	1,3,5,6-tetra-O-acetyl-2,4-di-O-methyl galactitol	379	1.75	87,101,118,129,160,189,234

RT, retention time; MW, molecular weight.

Further, based on the information in HSQC spectra (Figure 4D), the cross-peak signals in anomeric region at δH 5.01/δC 99.59, δH 4.90/δC 100.44, δH 4.58/δC 104.31, δH 4.89/δC 100.34, δH 5.06/δC 100.51, δH 4.39/δC 103.39, δH 4.48/δC 102.88, δH 4.43/δC 103.23, δH 5.03/δC 107.42, δH 5.19/δC 109.26, and δH 4.46/δC 104.86 were signed as → 4)-α-D-Gal*p*A-(1 → (A), → 4)-α-D-Gal*p*A-6-OMe-(1 → (B), → 4)-β-D-Gal*p*-(1 → (C), α-D-Gal*p*A-(1 → (D), → 4,6)-α-D-Glc*p*-(1 → (E), β-D-Xyl*p*-(1 → (F), β-D-Gal*p*-(1 → (G), → 4)-β-D-Glc*p*-(1 → (H), → 5)-α-L-Ara*f*-(1 → (I), α-L-Ara*f*-(1 → (J), → 3,6)-β-D-Gal*p*-(1 → (K), and → 2,4)-α-L-Rha*p*-(1 → (L), respectively. Based on the ^1^H, ^13^C, and ^1^H–^1^H COZY spectra (Figures 4A–C), the chemical shifts of all hydrogen and carbon atoms in different residues were assigned and shown in Table 2.

**Figure 4:**
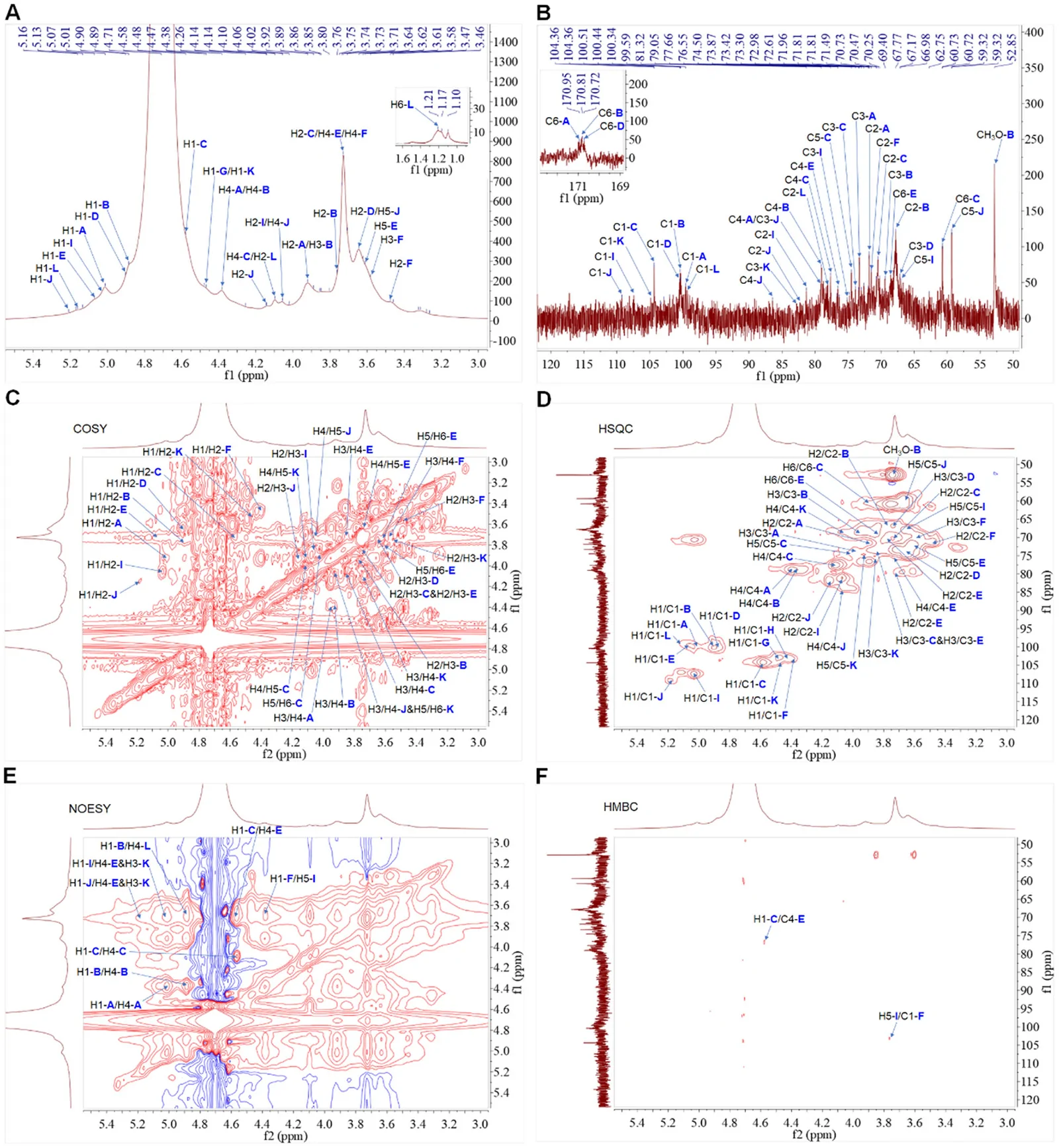
The NMR analysis (500 MHz) of BBPP80–1a polysaccharide at 25°C in D_2_O solution with a concentration of 40 mg/mL. **(A)** selected region of ^1^H NMR spectrum; **(B)** Selected region of ^13^C NMR spectrum; **(C)** ^1^H–^1^H COZY spectrum; **(D)** HSQC spectrum; **(E)** NOESY spectrum; **(F)** HMBC spectrum. (The blue arrows pointed out the hydrogen signals, carbon signals, corresponding hydrogen-hydrogen signals, carbon-hydrogen signals within and between residues).

**Table 2:** The chemical shift and corresponding assignments of ^1^H and ^13^C data for BBPP80–1a polysaccharide.

Code	Residues	Chemical shifts (ppm)						
		H1/C1	H2/C2	H3/C3	H4/C4	H5/C5	H6/C6	CH_3_O
A	→ 4)-α-D-Gal*p*A-(1 →	5.01	3.92	3.97	4.38	/	/	/
		99.59	71.90	73.29	79.08	/	170.96	/
B	→ 4)-α-D-Gal*p*A-6-OMe-(1 →	4.90	3.78	3.94	4.36	/	/	3.74
		100.44	67.69	69.39	78.98	/	170.81	52.85
C	→ 4)-β-D-Gal*p*-(1 →	4.58	3.73	3.85	4.10	4.02	3.91	/
		104.31	70.45	73.83	77.67	74.47	60.70	/
D	α-D-Gal*p*A-(1 →	4.89	3.65	3.75	/	/	/	/
		100.34	73.62	67.17	/	/	170.72	/
E	→ 4,6)-α-D-Glc*p*-(1 →	5.06	3.76	3.85	3.73	3.62	3.74, 3.82	/
		100.51	70.93	73.70	76.52	72.41	68.56	/
F	β-D-Xyl*p*-(1 →	4.39	3.49	3.58	3.72	3.74	/	/
		103.39	71.44	70.93	70.24	67.71	/	/
G	β-D-Gal*p*-(1 →	4.48	/	/	/	/	/	/
		102.88	/	/	/	/	/	/
H	→ 4)-β-D-Glc*p*-(1 →	4.43	/	/	3.86	/	/	/
		103.23	/	/	76.93	/	/	/
I	→ 5)-α-L-Ara*f*-(1 →	5.03	4.06	3.83	/	3.67, 3.76	/	/
		107.42	81.32	75.14	/	66.98	/	/
J	α-L-Ara*f*-(1 →	5.19	4.15	3.93	4.06	3.65, 3.77	/	/
		109.26	81.92	79.08	85.41	59.27	/	/
K	→ 3,6)-β-D-Gal*p*-(1 →	4.46	3.55	3.73	4.11	3.92	4.03	/
		104.86	70.09	80.22	69.19	73.76	70.83	/
L	→ 2,4)-α-L-Rha*p*-(1 →	5.12	4.11	/	3.68	/	1.21	/
		99.33	77.82	/	79.58	/	16.44	/

The cross-peak signals between H1 position of residue A and H4 position of residue A, and H1 position of residue B and H4 position of residue B at δH 5.01/δH 4.38 and δH 4.90/δH 4.36 ppm (Figure 4E), respectively, indicated → 4)-α-D-Gal*p*A-(1 → and → 4)-α-D-Gal*p*A-6-OMe-(1 → were connected to each other through 1 → 4 glycosidic linkage. Similarly, the cross-peak signal at δH 4.58/δH 4.10 ppm suggested that → 4)-β-D-Gal*p*-(1 → unit connected another → 4)-β-D-Gal*p*-(1 → residue by 1 → 4 glycosidic linkage. The cross-peaks between position-4 of residue E and position-1 of residue C at δH 4.58/δH 3.73 ppm (Figure 4E) and δH 4.58/δC 76.52 ppm (Figure 4F) suggested that → 4)-β-D-Gal*p*-(1 → unit had a 1 → 4 glycosidic linkage with → 4,6)-α-D-Glc*p*-(1 → residue. In addition, the signal between H1 position of sugar residue B and H4 position of residue L at δH 4.90/δH 3.68 ppm (Figure 4E) illustrated that there was a 1 → 4 glycosidic linkage between → 4)-α-D-Gal*p*A-6-OMe-(1 → unit and → 2,4)-α-L-Rha*p*-(1 → residue. Based on these methylation results (Table 1), NMR data (Figure 4; Table 2), and previous studies (16), BBPP80–1a was determined as a pectin polysaccharide whose homogalacturonan (HG) region was consisted of → 4)-α-D-Gal*p*A-(1 → and → 4)-α-D-Gal*p*A-6-OMe-(1 → units, and a small amount of → 2,4)-α-L-Rha*p*-(1 → unit. The micromorphology analysis (Supplementary Figure S4) showed BBPP80–1a had a highly porous, reticular, and heterogeneous microstructure, constructed from interwoven, flexible, ribbon-like or lamellar structures.

### Myocardial injury protection mechanism

In Figure 5A, cell viability assays indicated that BBPP80–1 fraction were non-toxic between 15.63 and 500 μg/mL (*p* > 0.05), while BBPP80–1a during 7.81 and 250 μg/mL did not decrease the cell viability in H9c2 cells (*p* > 0.05). After H/R modeling, the cell viability significantly reduced (*p* < 0.001). Dexrazoxane (10 μM), BBPP80–1 fraction (31.25–500 μg/mL) and BBPP80–1a (15.63–250 μg/mL) all obviously elevated the cell viability (*p* < 0.05, Figure 5B). Similarly, the levels of CK-MB and LDH both notably increased after H/R modeling (*p* < 0.001, Figure 5C). BBPP80–1 fraction (31.25 and 125 μg/mL) and BBPP80–1a (15.63 and 62.5 μg/mL) both could lower their levels (*p* < 0.05, Figure 5C). Flow cytometry analysis results suggested that many H9c2 cells underwent apoptosis (about 15-fold) after H/R modeling (*p* < 0.001, Figure 5D). Co-incubation with BBPP80–1 fraction and BBPP80–1a both could obviously reduce the level of cell apoptosis (*p* < 0.05, Figure 5D). The apoptosis related proteins including cleaved caspase 3 and BAX both significantly increased after H/R modeling (*p* < 0.001, Figure 5E), while anti-apoptotic protein BCL-2 exhibited a downward trend (*p* < 0.001, Figure 5E). BBPP80–1 fraction (125 μg/mL) and BBPP80–1a (15.63 and 62.5 μg/mL) both can reverse these abnormal protein expressions (*p* < 0.05, Figure 5E), which kept in line with the flow cytometry analysis results (Figure 5D).

**Figure 5:**
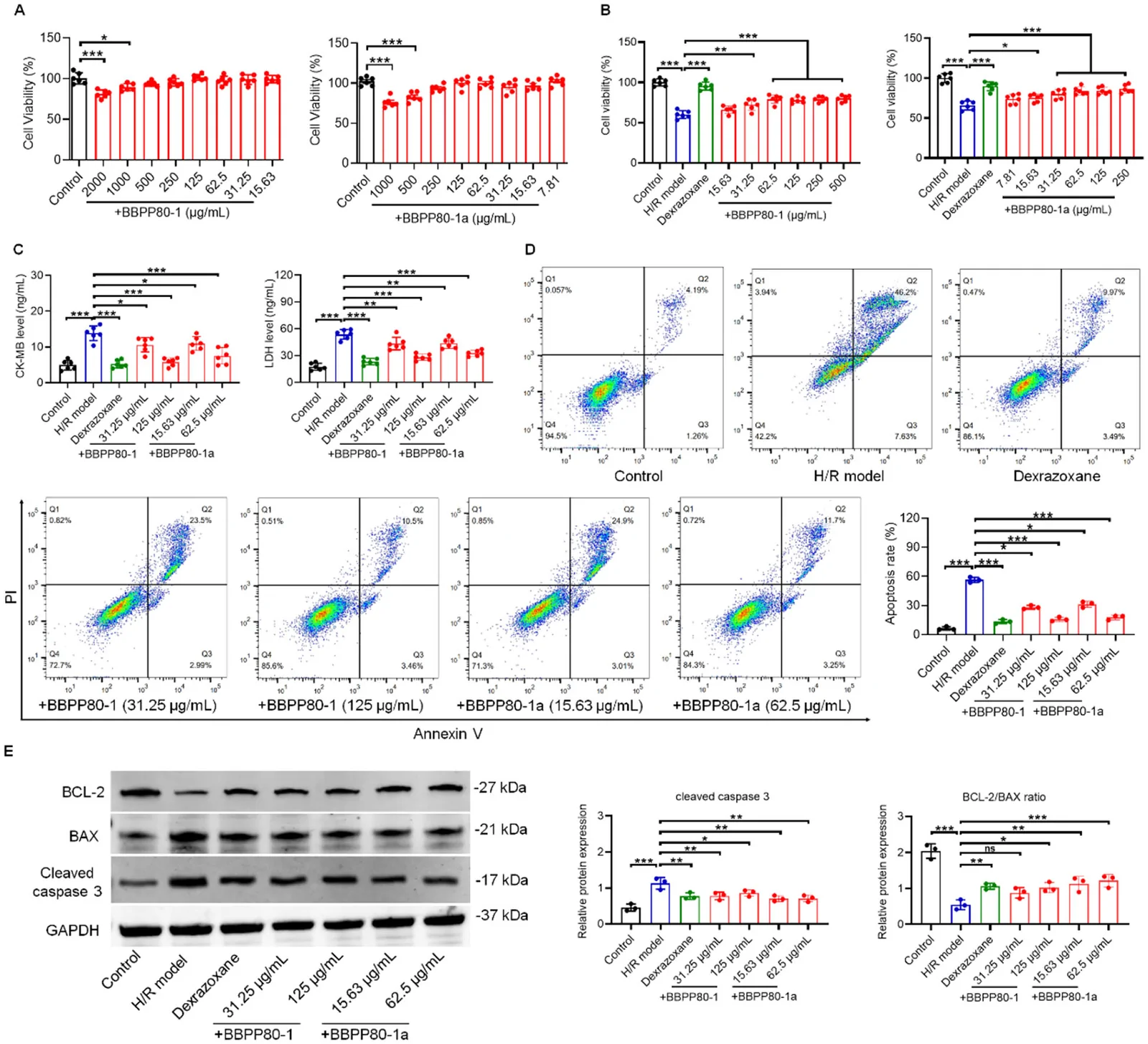
BBPP80–1 and BBPP80–1a polysaccharide improved the apoptosis of H9c2 cells in H/R-induced cell injury. **(A)** Cell viabilities after BBPP80–1 and BBPP80–1a were treated with H9c2 cells, respectively. (*n* = 6, One-way ANOVA followed by Tukey post-hoc test, * compared with those in control group, * *p* < 0.05, ** *p* < 0.01, *** *p* < 0.001). **(B)** Protection activities of BBPP80–1 and BBPP80–1a on H/R-induced H9c2 cell injury (*n* = 6); **(C)** Effects of BBPP80–1 and BBPP80–1a on CK-MB and LDH levels (*n* = 6); **(D)** Representative images and apoptosis ratio of total apoptotic cells (Q2 and Q3) stained by Annexin V-FITC/PI in control, H/R model, dexrazoxane (10 μM), BBPP80–1 (31.25 and 125 μg/mL), and BBPP80–1a (15.63 and 62.5 μg/mL) (*n* = 3); Q2 and Q3 represent late apoptosis and early apoptosis, respectively; **(E)** Immunoblot images and relative ratios of cleaved caspase 3, BCL-2, and BAX in H/R-induced H9c2 cells (*n* = 3); (One-way ANOVA followed by Tukey *post-hoc* test, * compared with the information in H/R model group, * *p* < 0.05, ** *p* < 0.01, *** *p* < 0.001).

Further, we explored the potential myocardial injury protection mechanism of BBPP80–1 fraction and BBPP80–1a through PI3K/AKT/mTOR and JNK/MAPK pathways. Notably, H/R modeling in H9c2 cells brought significant decreases of the phosphorylation expression of *p*-PI3K, *p*-AKT, and *p*-mTOR (*p* < 0.001, Figure 6A). When the cells were incubated with BBPP80–1 fraction (31.25 and 125 μg/mL) and BBPP80–1a (15.63 and 62.5 μg/mL), the ratios of *p*-PI3K/PI3K, *p*-AKT/AKT, and *p*-mTOR/mTOR all increased to a certain extent (*p* < 0.05, Figure 6A). Similarly, H/R modeling caused significant increase of *p*-JNK level (*p* < 0.001) and *p*-MAPK level (*p* < 0.05) in H9c2 cells (Figure 6B). BBPP80–1 fraction (31.25 and 125 μg/mL) and BBPP80–1a (62.5 μg/mL) can remarkably reduce the phosphorylation level of *p*-JNK (*p* < 0.05, Figure 6B). BBPP80–1a (15.63 and 62.5 μg/mL) brought decreased expression of *p*-MAPK level (*p* < 0.05, Figure 6B).

**Figure 6:**
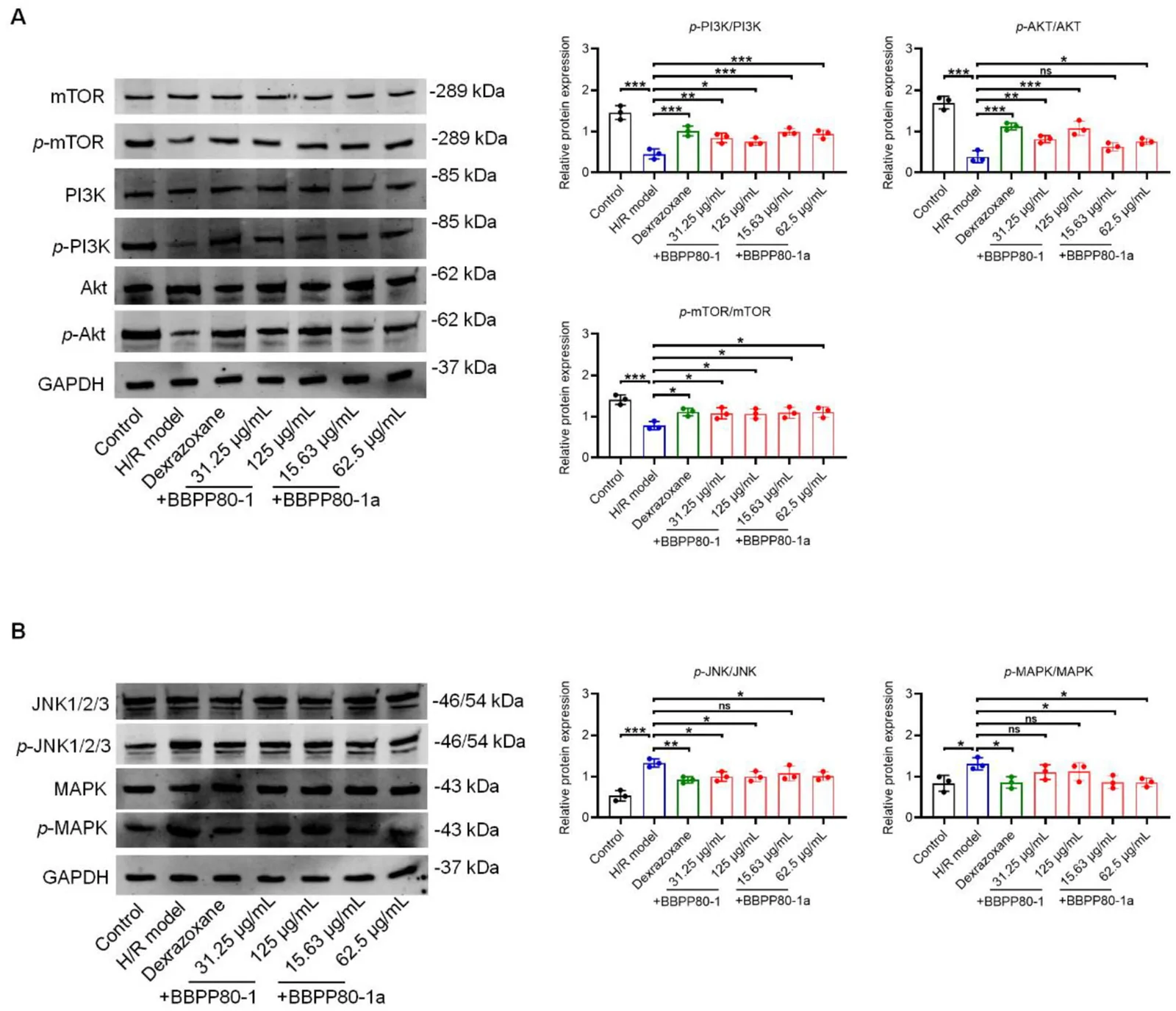
The regulatory effects of BBPP80–1 and BBPP80–1a polysaccharide toward PI3K/AKT/mTOR and JNK/MAPK pathways in H9c2 cells. **(A)** western blotting bands and relative ratios of key proteins in PI3K/AKT/mTOR pathways (*n* = 3); **(B)** protein expression and relative ratios of key targets in JNK/MAPK pathways (*n* = 3). (One-way ANOVA followed by Tukey post-hoc test, * compared with the information in H/R model group, * *p* < 0.05, ** *p* < 0.01, *** *p* < 0.001).

To verify the important roles of PI3K/AKT/mTOR and JNK/MAPK pathways in BBPP80–1a-related myocardial injury protective effects, LY294002 (PI3K inhibitor), SP600125 (JNK inhibitor) and SB203580 (MAPK inhibitor) were used to investigate the cell viability and apoptosis level. After LY294002 (10 μM) was used in H/R-induced H9c2 cells, cell viability obviously decreased (*p* < 0.001, Supplementary Figure S5A) and apoptosis level significantly increased (*p* < 0.001, sSupplementary Figure S5B) compared with those in H/R group. Meanwhile, the phosphorylation levels of *p*-PI3K, *p*-AKT and *p*-mTOR all notably reduced (*p* < 0.05, Figure 7A) compared with those in H/R group. When BBPP80-1a (62.5 μg/mL) were added in H/R + LY294002 group, the protein levels of *p*-PI3K, *p*-AKT and *p*-mTOR all notably increased (*p* < 0.05, Figure 7A). The cell viability significantly elevated (*p* < 0.001, Supplementary Figure S5A), and apoptosis level obviously decreased (*p* < 0.001, Supplementary Figure S5B).

**Figure 7:**
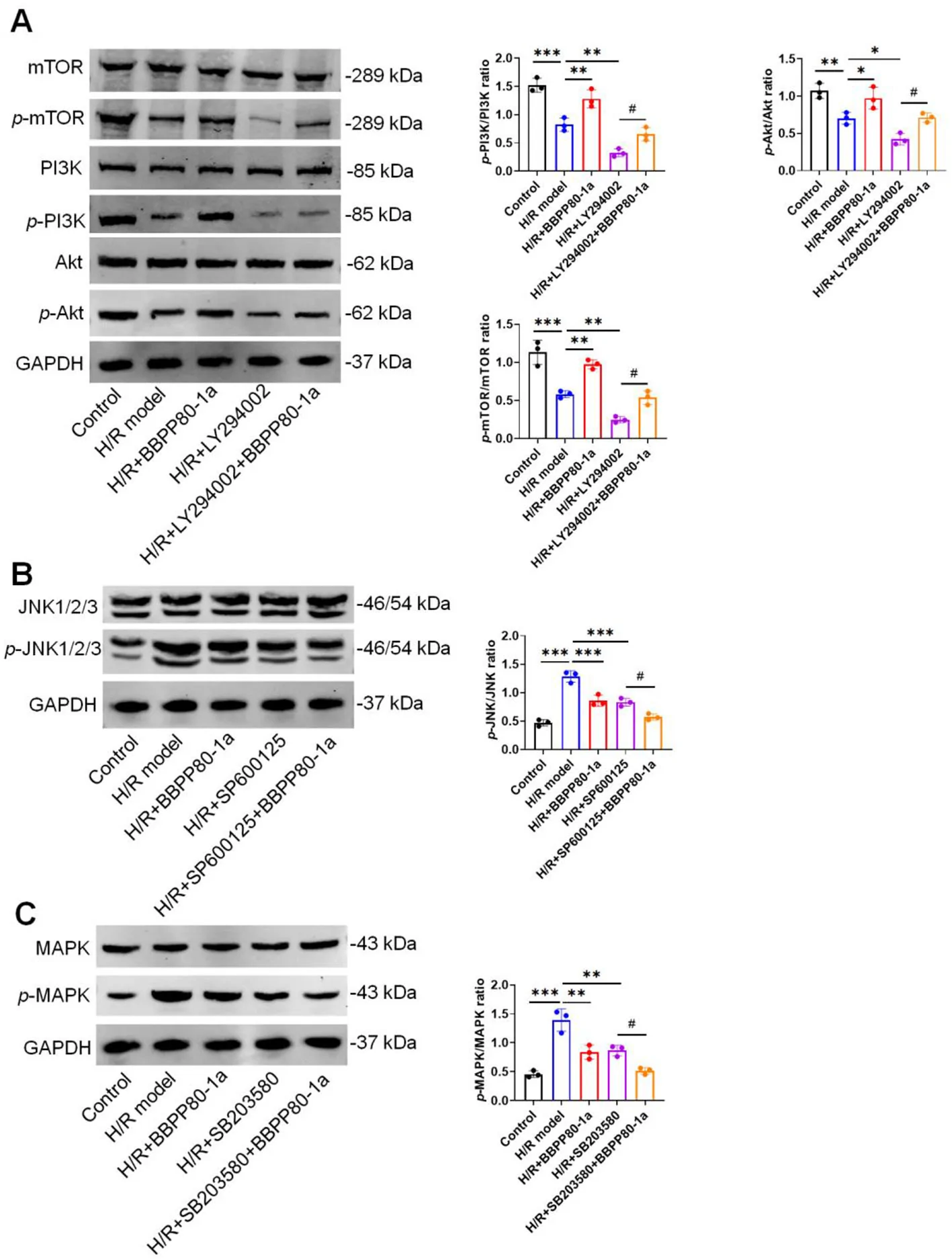
The potential myocardial injury protection mechanism of BBPP80–1a polysaccharide in H9c2 cells. **(A)** Protein levels and relative ratio of key proteins in PI3K/AKT/mTOR pathways in control, H/R model, H/R + BBPP80–1a (62.5 μg/mL) group, H/R + LY294002 (10 μM) group, H/R + LY294002 (10 μM) + BBPP80–1a (62.5 μg/mL) group (*n* = 3). **(B)** Protein expression and relative levels of JNK and *p*-JNK protein in control, H/R model, H/R + BBPP80–1a (62.5 μg/mL) group, H/R + SP600125 (10 μM) group, H/R + SP600125 (10 μM) + BBPP80–1a (62.5 μg/mL) group (*n* = 3). **(C)** Phosphorylation expression and relative ratio of *p*-38-MAPK in control, H/R model, H/R + BBPP80–1a (62.5 μg/mL) group, H/R + SB203580 (5 μM) group, H/R + SB203580 (5 μM) + BBPP80–1a (62.5 μg/mL) group (*n* = 3). (One-way ANOVA followed by Tukey *post-hoc* test, * compared with the information in H/R model group; ^#^ compared with the information in H/R + inhibitors group; * *p* < 0.05, ** *p* < 0.01, *** *p* < 0.001; ^#^ *p* < 0.05).

Similarly, SP600125 (10 μM) also could bring increased cell viability (*p* < 0.001, Supplementary Figure S6A) and decreased apoptosis level (*p* < 0.05, Supplementary Figure S6B) in H/R-induced H9c2 cells. The protein level of *p*-JNK also significantly reduced (*p* < 0.001, Figure 7B) compared with that in H/R group. After BBPP80-1a (62.5 μg/mL) was co-incubated in H/R + SP600125 group, the protein expression of *p*-JNK further declined (*p* < 0.05, Figure 7B) as well as increased cell viability (*p* < 0.001, Supplementary Figure S6A) and decreased cell apoptosis (*p* < 0.001, Supplementary Figure S6B).

Moreover, SB203580 (5 μM) led to significant decrease of *p*-MAPK protein level (*p* < 0.01, Figure 7C) and apoptosis level (*p* < 0.01, Supplementary Figure S7B) compared with those in H/R-induced H9c2 cells. The cell viability got obvious increase (*p* < 0.001, Supplementary Figure S7A). BBPP80-1a (62.5 μg/mL) brought further decrease of *p*-MAPK protein expression (*p* < 0.05, Figure 7C) and apoptosis level (*p* < 0.001, Supplementary Figure S7B) compared with those in H/R + SB203580 group. The cell viability also significantly raised (*p* < 0.001, Supplementary Figure S7A) compared with that in H/R + SB203580 group. This evidence illustrated that BBPP80–1a may increase cell vitality and reduce apoptosis level in H/R-induced H9c2 cells through PI3K/AKT/mTOR and JNK/MAPK pathways.

## Discussion

In this study, a novel acidic polysaccharide (BBPP80–1a) was successfully isolated and purified from active BBPP80–1 fraction of blueberry pomace. Its structural characterization indicated that BBPP80–1a was one high-homogalacturonan type pectin which was primarily composed of galacturonic acid along with significant amounts of galactose, arabinose, glucose, xylose, and rhamnose (Supplementary Table S2; Table 1). The methylation results (Table 1) suggested that its backbone was predominantly constructed by repetitive → 4)-α-D-Gal*p*A-(1 → and → 4)-α-D-Gal*p*A-6-OMe-(1 → units and a small amount of → 2,4)-α-L-Rha*p*-(1 → unit, indicating the existence of both HG and rhamnogalacturonan I (RG-I) domains in BBPP80–1a. Furthermore, the presence of various terminal and branched units of galactose and arabinose illustrated that BBPP80–1a was with highly branched and complex structures. In our previous study (16), BBPP80–2b was an acidic polysaccharide also from blueberry pomace. Its contents of galacturonic acid were 9.04% and 8.29% in monosaccharide composition and methylation results, respectively. Compared with BBPP80–2b, BBPP80–1a showed a more typical acidic pectin character and a more pronounced HG backbone, with a higher galacturonic acid content of 50.92% and 48.01% in methylation analysis (Table 1) and monosaccharide analysis (Supplementary Table S2), respectively. Compared with previous studies, BBPP80–1a also showed significant differences of HG and RG-I ratios (Supplementary Table S2) with acidic polysaccharides from blueberry lyophilized powder (34). These structural differences, particularly the ratio of HG/RG-I and the degree of branching, were likely the determinants of its unique biological activity (32, 34, 35).

The *in vitro* cardioprotective evaluation results indicated that BBPP80–1a can significantly improve the viability of H9c2 cardiomyocytes subjected to H/R injury, reduce the release of LDH and CK-MB, and effectively inhibit H/R-induced apoptosis (Figures 5A–D). Further protein expression results revealed that BBPP80–1a can regulate the levels of cleaved caspase 3, BAX and BCL-2 (Figure 5E). The mechanistic investigation showed that BBPP80–1a (62.5 μg/mL) obviously upregulated the phosphorylation levels of PI3K, AKT, and mTOR (Figure 7A) after LY294002 (10 μM) were co-incubated with H9c2 cells in H/R-induced cell injury model. Similarly, the phosphorylation expression of JNK, and p38-MAPK also altered after BBPP80–1a (62.5 μg/mL) were co-incubated with SP600125 (or SB203580) in H/R-induced H9c2 cell injury model (Figures 7B, C). These findings suggested that the cardioprotective effect of BBPP80–1a was achieved through the activation of PI3K/AKT/mTOR survival signaling pathway and the regulation of the JNK/MAPK stress signaling pathways. This regulatory pattern was highly consistent with the cardioprotective mechanisms of various plant-derived polysaccharides (17, 22, 24). For example, polysaccharides from *Stropharia rugosoannulata* can ameliorate ventricular remodeling and reduce cardiomyocyte apoptosis in rats with heart failure by activating the PI3K/AKT/mTOR pathway (22), while polysaccharides derived from *Phellinus linteus* can protect H9c2 cells from H/R injury through modulation of the PI3K/AKT pathway (17). *Astragalus polysaccharides* also can reduce *p*-JNK and *p*-MAPK levels in diabetic cardiomyopathy mice, thereby ameliorating cardiac dysfunction (24). These results further indicated BBPP80–1a may promote cardiomyocyte survival by balancing these two competitive signaling pathways, and confirmed the potential of blueberry pomace polysaccharides in ameliorating H/R-induced injury in cardiomyocytes.

The significant biological activity of BBPP80–1a may be corresponded to its unique chemical structure. In the current study, the high content of galacturonic acid and the HG-rich backbone in BBPP80–1a (Table 1) likely represented one of the key structural bases for its activity (32, 36). The HG and RG-I ratios of BBPP80–1a were 44.49% and 41.35%, respectively (Supplementary Table S2). Previous reports displayed that pectins with high content of galacturonic acid were prone to interact with receptors on cell surface, further triggering downstream intracellular signaling cascades (32, 37). In addition, the RG-I domains and diverse galactan and arabinan side chains in BBPP80–1a may provide more recognition sites of specific receptors on the cell membrane (38, 39). The molecular weight (*M*_w_ = 164.91 kDa, Supplementary Table S1) and branched structures (Table 1) of BBPP80–1a may play an important role in the activation of PI3K/AKT and MAPK pathways (40, 41). The porous network structure observed by SEM (Supplementary Figure S4) may facilitate endocytosis for direct intracellular signaling protein interaction (42). Although the activity of polysaccharides with different molecular weights or branched structures were not directly compared, we reasonably hypothesized that the unique HG/RG-I core and fine branching structure of BBPP80–1a were critical for its effective activation of the downstream pathways, thereby realizing cardioprotection effects (42, 43).

This study had successfully characterized a novel acidic pectic polysaccharide from BBPP80–1 fraction of blueberry pomace, which exhibited significant cardioprotective activity (Figure 5). BBPP80–1a can inhibit the cardiomyocyte apoptosis *via* PI3K/AKT/mTOR and JNK/MAPK pathways (Figures 6, 7). This work not only provided a new strategy for the high-value utilization of blueberry pomace, but also offered a crucial theoretical basis for developing natural functional agents for the prevention and treatment of myocardial I/R injury. Nevertheless, there were some limitations for this study. For instance, the activity and mechanistic studies of BBPP80–1a have only been conducted at the cellular level. The *in vivo* cardioprotective efficacy of BBPP80–1a in animal models has not been performed yet. The specific targets directly interacted with by BBPP80–1a also remain unclear and unidentified. Furthermore, it remains to be seen that the oral bioavailability and *in vivo* metabolic fates of BBPP80–1a can be explored. In future study, the work should concentrate on investigating its efficacy and safety using *in vivo* models, and determining its specific molecular targets using proteomics and surface plasmon resonance approach. This work would benefit the application of blueberry pomace polysaccharides as functional foods or nutraceuticals for the prevention and treatment of cardiovascular diseases.

## Conclusion

In this study, a purified polysaccharide, BBPP80–1a, was prepared from the BBPP80–1 fraction of blueberry pomace. Galacturonic acid was the most abundant monosaccharide and occupied about 50% in the composition of BBPP80–1a. Monosaccharide composition analysis, methylation results, and NMR data confirmed that BBPP80–1a was determined as a high-homogalacturonan type pectin polysaccharide with highly branched and complex structures. Micromorphology analysis suggested that it was with a highly porous, reticular, and heterogeneous microstructure. The mechanistic investigation indicated that BBPP80–1a played an important role in cardioprotective effect through PI3K/AKT/mTOR and JNK/MAPK pathways. This study not only provided a new active component for developing blueberry pomace-based functional foods or pharmaceuticals, but also offered a scientific basis for high-value utilization of agricultural by-products, in line with circular economy and sustainable development concepts.

## Data Availability

The original contributions presented in the study are included in the article/Supplementary material, further inquiries can be directed to the corresponding authors.
