# Supplementary material for: Structural characterization and in vitro myocardial injury protection potential of one acidic polysaccharide from blueberry (Vaccinium spp.) pomace
Source: Front Nutr. 2026 Sep 22;13:1920321. doi: 10.3389/fnut.2026.1920321 (PMC13638605; doi:10.3389/fnut.2026.1920321)
Supplement: Supplementary file 1 [file Supplementary_file_1.docx]

**Supporting information**

**Structural characterization and *in vitro* myocardial injury protection potential of one acidic polysaccharide from blueberry (*Vaccinium spp.*) pomace**

Beibei Zhang ^1†^, Qiwen Zhang ^1†^, Ning Huang ^1^, Mengmeng Jia ^1^, Han Xing ^1^, Yongjie Yang ^1^, Dinglei Xue ^2^, Jing Yang ^1^*, Zifei Qin ^1^*

^1^ Department of Pharmacy, Henan Province Engineering Research Center of Application & Translation of Precision Clinical Pharmacy, the First Affiliated Hospital of Zhengzhou University, Zhengzhou 450052, China;

^2^ Jiangsu Sanshu Biotechnology Co., Ltd., Nantong 226006, China;

^†^These authors contributed equally to this work.

***Corresponding Authors:**

Dr. Zifei Qin, qzf1989@163.com

Dr. Jing Yang, jingyang_0101@163.com;

**Results**

**Structural characterization of BBPP80–1a**

**UV–vis analysis**

As shown in UV–vis spectrum of BBPP80–1a (Fig. 2C), there were no observable absorption peaks at 260, 280, and 620 nm, indicating that it had low or undetectable levels of nucleic acids, proteins, and pigments.

**FT–IR analysis**

In Fig. 2D, the absorption band at 3436.12 cm^-1^ was attributed to the O–H stretching vibrations associated with intermolecular or intramolecular hydrogen bonding. The band for asymmetric C–H stretching vibrations from CH, CH2, and CH3 groups was observed at 2925.21 cm^-1^. The band at 1633.94 cm^-1^ (Fig. 2D) corresponded to the asymmetric stretching of carboxyl groups (-COOH or -COO^-^), while the band at 1401.88 cm^-1^ (Fig. 2D) was considered as the symmetric stretching vibration of carboxyl groups (-COOH or -COO^-^). The bands for stretching vibration of C–O–H and C–O–C structural arrangements were detected at 1103.50 and 1018.79 cm^-1^.

**Molecular weight determination**

The gel permeation chromatography-refractive index multi-angle laser light scattering detection profile (GPC-MALLS), molecular conformation diagram, and high-performance gel permeation chromatography (HPGPC) of BBPP80–1a were shown in Fig. S1. Meanwhile, the parameters including molar mass (g/mol), polydispersity and root mean square radius (nm) were displayed in Table S1. The *M*_w_ and *M*_n_ distributions were 164.91 and 78.43 kDa, respectively. The polydispersity (*M*_w_/*M*_n_) value was 2.10, which indicated that BBPP80–1a was a relatively homogeneous polysaccharide. This kept in line with its purity of 91.7%. In addition, the root mean square radii were all less than 29.0 nm.

**Monosaccharide composition analysis**

The ion chromatogram of BBPP80–1a was shown in Fig. S2. Quantitative results (Table S2) suggested that galacturonic acid and galactose were the most abundant monosaccharides which accounted for 48.01% and 22.29%, respectively. Arabinose and glucose also occupied for 12.02% and 9.36%, respectively. The molar ratio of xylose and rhamnose were 4.46% and 3.52%, respectively. Obviously, it was an acidic polysaccharide.

**Methylation and GC–MS analysis**

The total ion chromatograms of mono-deuterated and double-deuterated BBPP80–1a samples by GC–MS were shown in Fig. 3. Every chromatographic peak had a respective mass spectrum (Fig. S3). Compared with their mass fragments and abundances with those in Complex Carbohydrate Research Center (CCRC) Spectral Database for Partially Methylated Alditol Acetates (PMAAs), these peaks were identified as different residues. Their detailed information was shown in Table 1. Obviously, the →4)-Gal*p*A-(1→ unit was the most abundant sugar residue with molar ratio of 43.62%, while →4)-Gal*p*-(1→ accounted for about 17.06%. Gal*p*A-(1→ occupied for 5.37%, while the ratio of other derivatives including →4,6)-Glc*p*-(1→, Xylp-(1→, Gal*p*-(1→, and →4)-Glc*p*-(1→ were all less than 5.0%.

For galacturonic acid, the total ratio of BBPP80–1a in methylation analysis (Table 1) and monosaccharide analysis (Table S2) were 50.92% and 48.01%, respectively. Similarly, the molar ratio of galactose in methylation analysis and monosaccharide analysis was 25.12% and 22.29%, respectively. The ratio of arabinose in these analysis results was 8.10% and 12.02%, respectively, while glucose accounted about 9.18% and 9.36% in these two results, respectively. Overall, the methylation results (Table 1) were basically consistent with the monosaccharide composition results (Table S2).

**NMR analysis**

In Fig. 4A, some signals between δH 4.3–5.4 ppm were observed in anomeric signal region, suggested that BBPP80–1a included many sugar residues. The signals of anomeric hydrogen were detected at δH 5.01, 4.90, 4.58, 4.89, 5.06, 4.39, 4.48, 4.43, 5.03, 5.19, and 4.46 ppm. Similarly, many signals were obviously present between δC 90–120 ppm in anomeric carbon region of ^13^C NMR spectra (Fig. 4B). Further, based on the information in HSQC spectra (Fig. 4D), the cross-peak signals in anomeric region at δH 5.01/δC 99.59, δH 4.90/δC 100.44, δH 4.58/δC 104.31, δH 4.89/δC 100.34, δH 5.06/δC 100.51, δH 4.39/δC 103.39, δH 4.48/δC 102.88, δH 4.43/δC 103.23, δH 5.03/δC 107.42, δH 5.19/δC 109.26, and δH 4.46/δC 104.86 were signed as residues A, B, C, D, E, F, G, H, I, J, K, and L, respectively. Compared with the methylation results and previous study, these units were characterized as →4)-α-D-Gal*p*A-(1→ (A), →4)-α-D-Gal*p*A-6-OMe-(1→ (B), →4)-β-D-Gal*p*-(1→ (C), α-D-Gal*p*A-(1→ (D), →4,6)-α-D-Glc*p*-(1→ (E), β-D-Xyl*p*-(1→ (F), β-D-Gal*p*-(1→ (G), →4)-β-D-Glc*p*-(1→ (H), →5)-α-L-Ara*f*-(1→ (I), α-L-Ara*f*-(1→ (J), →3,6)-β-D-Gal*p*-(1→ (K), and →2,4)-α-L-Rha*p*-(1→ (L), respectively. In ^1^H–^1^H COSY spectra (Fig. 4C), the cross peak at δH 5.01/δH 3.92 ppm indicated that the chemical shift of H-2 in residue A was at δH 3.92 ppm. Based on the cross-peak signal at δH 3.92/δC 71.90 ppm in Fig. 4D, the chemical shift of C-2 in residue A was determined at δC 71.90 ppm. Similarly, the chemical shifts of other hydrogen and carbon atoms in different residues were assigned and shown in Table 2.

The information in NOESY spectra (Fig. 4E) and HMBC spectra (Fig. 4F) could well present the linkage patterns of these different units. Two obvious cross-peak signals between H1 position of residue A and H4 position of residue A, and H1 position of residue B and H4 position of residue B at δH 5.01/δH 4.38 and δH 4.90/δH 4.36 ppm (Fig. 4E), respectively, indicated →4)-α-D-Gal*p*A-(1→ and →4)-α-D-Gal*p*A-6-OMe-(1→ were connected to each other through 1→4 glycosidic linkage. Similarly, the cross-peak signal at δH 4.58/δH 4.10 ppm suggested that →4)-β-D-Gal*p*-(1→ unit connected another →4)-β-D-Gal*p*-(1→ residue by 1→4 glycosidic linkage. The signals between position-5 of residue I and position-1 of residue F at δH 4.39/δH 3.76 ppm (Fig. 4E) and δH 3.76/δC 103.39 ppm (Fig. 4F) indicated that β-D-Xyl*p*-(1→ reside connected to →5)-α-L-Ara*f*-(1→ unit through 1→4 glycosidic linkage. The cross-peaks between position-4 of residue E and position-1 of residue C at δH 4.58/δH 3.73 ppm (Fig. 4E) and δH 4.58/δC 76.52 ppm (Fig. 4F) suggested that →4)-β-D-Gal*p*-(1→ unit had a 1→4 glycosidic linkage with →4,6)-α-D-Glc*p*-(1→ residue. In addition, the signal between H1 position of sugar residue B and H4 position of residue L at δH 4.90/δH 3.68 ppm illustrated that there was a 1→4 glycosidic linkage between →4)-α-D-Gal*p*A-6-OMe-(1→ unit and →2,4)-α-L-Rha*p*-(1→ residue. The signals at δH 5.03/δH 3.73 ppm and δH 5.19/δH 3.73 ppm pointed that the position-1 of →5)-α-L-Ara*f*-(1→ and α-L-Ara*f*-(1→ units could be connected to the position-4 of →4,6)-α-D-Glc*p*-(1→ or position-3 of →3,6)-β-D-Gal*p*-(1→ residues. For some residues, neither clear nor reliable inter-residue correlation signals were detected in the HMBC (Fig. 4F) and NOESY (Fig. 4E) spectra, bring huge obstacles for their linkage patterns.

Based on these methylation results (Table 1), NMR data (Fig. 4 & Table 2), and previous studies (16), BBPP80–1a was determined as a pectin polysaccharide. The homogalacturonan (HG) region that was consisted of →4)-α-D-Gal*p*A-(1→ and →4)-α-D-Gal*p*A-6-OMe-(1→ units, and a small amount of →2,4)-α-L-Rha*p*-(1→ unit were the backbone of BBPP80–1a. The repetitive →4)-Gal*p*-(1→ unit mainly formed its branched chain. The α-D-Gal*p*A-(1→, →4,6)-α-D-Glc*p*-(1→, →4)-β-D-Glc*p*-(1→ and β-D-Gal*p*-(1→ also shared a certain contribution to branched chain of BBPP80–1a. This study mainly analyzed the reliable connection sites between different residues based on the clear and reliable evidence. Therefore, the overall repeating units or the complete saccharide chain sequence of BBPP80–1a were not further integrated or deduced.

**Micromorphology analysis**

The surface morphology of BBPP80–1a was shown in Fig. S4. At lower magnification (200×), it appeared as a dense, porous network structure formed by the interweaving and stacking of many fiber-like or sheet-like structures of varying thickness. Its surface was rough and exhibited a high degree of heterogeneity. As the magnification increased (500× and 2000×), it became apparent that the fibrous network contained many irregular pores of varying sizes, which were unevenly distributed. At 10000× magnification, the "threads" comprising the network were revealed to be broad, flat, ribbon-like or lamellar structures with curled edges. These lamellae connected and fold into each other to form a complex. Overall, BBPP80–1a exhibited a highly porous, reticular, and heterogeneous microstructure, constructed from interwoven, flexible, ribbon-like or lamellar structures.

**Table list**

**Table S1** The molecular weight, polydispersity, and root mean square of purified BBPP80-1a polysaccharide.

**Table S2** The monosaccharide composition and structural characteristics of purified BBPP80-1a polysaccharide.

**Figure captions**

**Figure S1** The molecular weight measurement result of BBPP80-1a polysaccharide. (A) The high-performance gel permeation chromatography-refractive index-multiangle laser light scattering (HPGPC-RI-MALS) chromatograph with molecular weight distribution of BBPP80-1a. (B) HPSEC-MALLS-RID chromatograms of BBPP80-1a polysaccharide. (C) HPGPC profiles of BBPP80-1a polysaccharide on Shodex SB-805 chromatographic columns. The variation tendencies of signals of the multi-angle laser scattering (LS), refractive index (RI), and fitted molar mass of LJPS. The red line indicates the variation tendency in the LS of LJPS with retention time, and the green line represents the trend in the RI of LJPS. The tendency of the red and green lines suggested the size of the PS molecules and their relative proportions contained in the tested sample. The blue line is the varying tendency of the molar mass fitted by the LS and RI signal of the PSs following the retention time.

**Figure S2** The monosaccharide composition analysis of BBPP80-1a polysaccharide (A), and reference standards (B). Each absorption peak was annotated based on the monomer name and retention time. Fucose, Fuc (*R*_t_: 4.27 min); Rhamnose, Rha (*R*_t_: 8.19 min); Arabinose, Ara (*R*_t_: 8.69 min); Galactose, Gal (*R*_t_: 10.94 min); Glucose, Glc (*R*_t_: 12.43 min); Xylose, Xyl (*R*_t_: 14.73 min); Mannose, Man (*R*_t_: 15.33 min); Fructose, Fru (*R*_t_: 18.18 min); Ribose, Rib (*R*_t_: 20.06 min); Galacturonic acid, Gal-UA (*R*_t_: 33.51 min); Glucuronic acid, Gul-UA (*R*_t_: 34.07 min); Guluronic acid, Glc-UA (*R*_t_: 35.38 min); Mannuronic acid, Man-UA (*R*_t_: 36.68 min).

**Figure S3** The mass fragments of different GC-MS chromatographic peaks from BBPP80-1a polysaccharide by EI mode. (A) mass fragments of peak 1 (*t*_R_ = 11.70 min) [Ara*f*-(1→]; (B) mass fragments of peak 2 (*t*_R_ = 12.73 min) [Xyl*p*-(1→]; (C) mass fragments of peak 2 (*t*_R_ = 14.51 min) [→3)-Ara*f*-(1→]; (D) mass fragments of peak 2 (*t*_R_ = 15.43 min) [→5)-Ara*f*-(1→]; (E) mass fragments of peak 3 (*t*_R_ = 15.62 min) [→2)-Xyl*p*-(1→]; (F) mass fragments of peak 4 (*t*_R_ = 17.44 min) [Gal*p*A-(1→]; (G) mass fragments of peak 3 (*t*_R_ = 17.46 min) [Gal*p*-(1→]; (H) mass fragments of peak 4 (*t*_R_ = 17.94 min) [→3,5)-Ara*f*-(1→]; (I) mass fragments of peak 5 (*t*_R_ = 18.36 min) [→2,4)-Rha*p*-(1→]; (J) mass fragments of peak 6 (*t*_R_ = 19.78 min) [→4)-Gal*p*A-(1→]; (K) mass fragments of peak 6 (*t*_R_ = 19.82 min) [→4)-Gal*p*-(1→]; (L) mass fragments of peak 6 (*t*_R_ = 19.92 min) [→2,3,5)-Ara*f*-(1→]; (M) mass fragments of peak 7 (*t*_R_ = 19.99 min) [→4)-Glc*p*-(1→]; (N) mass fragments of peak 8 (*t*_R_ = 20.27 min) [→3)-Gal*p*-(1→]; (O) mass fragments of peak 6 (*t*_R_ = 20.49 min) [→6)-Glc*p*-(1→]; (P) mass fragments of peak 9 (*t*_R_ = 21.50 min) [→6)-Gal*p*-(1→]; (Q) mass fragments of peak 10 (*t*_R_ = 21.91 min) [→3,4)-Gal*p*A-(1→]; (R) mass fragments of peak 6 (*t*_R_ = 23.28 min) [→4,6)-Glc*p*-(1→]; (S) mass fragments of peak 6 (*t*_R_ = 24.32 min) [→3,6)-Gal*p*-(1→].

**Figure S4** Surface morphology of BBPP80-1a polysaccharide by scanning electron microscope analysis at 200× (100 μM), 500× (40 μM), 2000× (10 μM), and 10000× (2 μM) scales, respectively.

**Figure S5** BBPP80–1a polysaccharide improved the cell viability and apoptosis levels after LY294002 was co-incubated with H9c2 cells in H/R-induced cell injury model. (A) cell viabilities of H9c2 cells in control, H/R model, H/R + BBPP80–1a (62.5 μg/mL) group, H/R + LY294002 (10 μM) group, H/R + LY294002 (10 μM) + BBPP80–1a (62.5 μg/mL) group (n = 6). (B) Representative images and apoptosis ratio of total apoptotic cells (Q2 and Q3) stained by Annexin V-FITC/PI in control, H/R model, H/R + BBPP80–1a (62.5 μg/mL) group, H/R + LY294002 (10 μM) group, H/R + LY294002 (10 μM) + BBPP80–1a (62.5 μg/mL) group (n = 3); Q2 and Q3 represent late apoptosis and early apoptosis, respectively. (One-way ANOVA followed by Tukey post-hoc test, * compared with the information in H/R model group; ^#^ compared with the information in H/R + LY294002 group; * *p <* 0.05, ** *p <* 0.01, *** *p <* 0.001; ^#^ *p <* 0.05, ^##^ *p <* 0.01, ^###^ *p <* 0.001).

**Figure S6** BBPP80–1a polysaccharide improved the cell viability and apoptosis levels after SP600125 was co-incubated with H9c2 cells in H/R-induced cell injury model. (A) cell viabilities of H9c2 cells in control, H/R model, H/R + BBPP80–1a (62.5 μg/mL) group, H/R + SP600125 (10 μM) group, H/R + SP600125 (10 μM) + BBPP80–1a (62.5 μg/mL) group (n = 6). (B) Representative images and apoptosis ratio of total apoptotic cells (Q2 and Q3) stained by Annexin V-FITC/PI in control, H/R model, H/R + BBPP80–1a (62.5 μg/mL) group, H/R + SP600125 (10 μM) group, H/R + SP600125 (10 μM) + BBPP80–1a (62.5 μg/mL) group (n = 3); Q2 and Q3 represent late apoptosis and early apoptosis, respectively. (One-way ANOVA followed by Tukey post-hoc test, * compared with the information in H/R model group; ^#^ compared with the information in H/R + SP600125 group; * *p <* 0.05, ** *p <* 0.01, *** *p <* 0.001; ^#^ *p <* 0.05, ^##^ *p <* 0.01, ^###^ *p <* 0.001).

**Figure S7** BBPP80–1a polysaccharide improved the cell viability and apoptosis levels after SB203580 was co-incubated with H9c2 cells in H/R-induced cell injury model. (A) cell viabilities of H9c2 cells in control, H/R model, H/R + BBPP80–1a (62.5 μg/mL) group, H/R + SB203580 (5 μM) group, H/R + SB203580 (5 μM) + BBPP80–1a (62.5 μg/mL) group (n = 6). (B) Representative images and apoptosis ratio of total apoptotic cells (Q2 and Q3) stained by Annexin V-FITC/PI in control, H/R model, H/R + BBPP80–1a (62.5 μg/mL) group, H/R + SB203580 (5 μM) group, H/R + SB203580 (5 μM) + BBPP80–1a (62.5 μg/mL) group (n = 3); Q2 and Q3 represent late apoptosis and early apoptosis, respectively. (One-way ANOVA followed by Tukey post-hoc test, * compared with the information in H/R model group; ^#^ compared with the information in H/R + SB203580 group; * *p <* 0.05, ** *p <* 0.01, *** *p <* 0.001; ^#^ *p <* 0.05, ^##^ *p <* 0.01, ^###^ *p <* 0.001).

**Table S1** The molecular weight, polydispersity, and root mean square of purified BBPP80–1a polysaccharide.

| **Item** |  | **BBPP80–1a** |
| --- | --- | --- |
| Molar mass (g/mol) | *M*_n_ (kDa) | 78.43 |
|  | *M*_p_ (kDa) | 105.65 |
|  | *M*_w_ (kDa) | 164.91 |
|  | *M*_z_ (kDa) | 529.73 |
| Polydispersity | *M*_w_/*M*_n_ | 2.10 |
|  | *M*_z_/*M*_n_ | 6.75 |
| Root means square radius, RMS (nm) | *R*_n_ (nm) | 27.3 |
|  | *R*_w_ (nm) | 27.2 |
|  | *R*_z_ (nm) | 29.0 |

**Table S2** The monosaccharide composition and structural characteristics of purified BBPP80-1a polysaccharide.

| **Monosaccharide** | **RT (min)** | **Slop** | **Fitting coefficient** | **Molar ratio (%)** |
| --- | --- | --- | --- | --- |
| Fucose (Fuc) | 4.27 | 1.2194 | 0.9916 | 0.34% |
| Rhamnose (Rha) | 8.19 | 1.3358 | 0.9994 | 3.52% |
| Arabinose (Ara) | 8.69 | 1.7974 | 0.9981 | 12.02% |
| Galactose (Gal) | 10.94 | 1.7359 | 0.9995 | 22.29% |
| Glucose (Glc) | 12.43 | 1.6828 | 0.9993 | 9.36% |
| Xylose (Xyl) | 14.73 | 1.4310 | 0.9965 | 4.46% |
| Mannose (Man) | 15.33 | 1.3278 | 0.9935 | 0.00% |
| Fructose (Fru) | 18.18 | 0.4340 | 0.9976 | 0.00% |
| Ribose (Rib) | 20.06 | 0.9699 | 0.9973 | 0.00% |
| Galacturonic Acid (Gal-UA) | 33.51 | 0.7587 | 0.9931 | 48.01% |
| Glucuronic Acid (Gul-UA) | 34.07 | 0.5295 | 0.9947 | 0.00% |
| Guluronic Acid (Glc-UA) | 35.38 | 1.0879 | 0.9979 | 0.00% |
| Mannuronic Acid (Man-UA) | 36.68 | 0.5052 | 0.9929 | 0.00% |
| HG (molar ratio, %) |  |  |  | 44.49 |
| RG-I (molar ratio, %) |  |  |  | 41.35 |
| Rha / GalA |  |  |  | 0.07 |
| (Gal +Ara) / Rha |  |  |  | 9.75 |

**Figure S1**

**Figure S2**

**Figure S3**

**Figure S4**

**Figure S5**

**Figure S6**

**Figure S7**
